# Measurement properties of the Knee Injury and Osteoarthritis Outcome Score Patello-Femoral questionnaire in Saudi Arabians

**DOI:** 10.7717/peerj.9323

**Published:** 2020-07-31

**Authors:** Mahamed Ateef

**Affiliations:** Department of Physical Therapy and Health Rehabilitation, College of Applied Medical Sciences, Majmaah University, Al Majmaah, Kingdom of Saudi Arabia

**Keywords:** Knee, Patello-femoral, Reliability, Validity, Psychometric, Arabic, Saudi Arabia

## Abstract

**Background:**

No Arabic or its dialect questionnaire is available to evaluate the anterior knee pain in the Saudi Arabian religious population. This study aims to translate, adapt, and psychometrically validate the Knee Injury and Osteoarthritis Outcome Score (KOOS-PF) Patellofemoral scale in the Arabic language in Saudi Arabic dialect.

**Method:**

Translation has been done as per standard guidelines. The questionnaire was administered to 95 patients to determine the psychometric properties including on two different occasions, with a 48-hour gap in-between; to ensure that their answers were reliable; 84 patients (88.4% compliance rate) responded for test and retest reliability, ceiling-floor effects, validity and other psychometric criteria.

**Results:**

Cronbach’s alpha (internal consistency) and test–retest reliability was good and excellent (∞ = 0.81; ICC > 0.95). None of the items showed >30% floor or ceiling effect and the minimal detectable change was within the acceptable range (<30%). The KOOS-PF subscale showed a moderate correlation (−0.568) with pain-visual analog scale for its construct validity.

**Conclusion:**

The Arabic dialect of KOOS-PFis reliable and valid to be used to evaluate isolated knee pain of patellofemoral origin in Muslim patients in Saudi Arabia.

## Introduction

Knee osteoarthritis (OA) is one among the ten disabling diseases affecting the mobility to 80% and activities of daily living to 25% (https://www.who.int/chp/topics/rheumatic/en/). The knee joint is composed of three compartments; patellofemoral joint (PFJ), a subgroup of the knee joint is also affected with arthritis together with tibiofemoral joint (TFJ) arthritis (Englund & Lohmander, 2005). One study by Hunter, March & Sambrook (2003) has found that the cartilage changes in Magnetic Resonance Imaging are related to increased pain symptoms as well as scores in subjective measures when compared to femorotibial symptoms.

Diffuse pain at the anteromedial aspect of the patella is a characteristic of PFJ arthritis that can be seen while running, squatting, ascending and descending stairs (McConnell, 1996; Powers, 1998). Among all the knee problems, PFJ pain accounts for 25% to 40% in the sports populace (McConnell, 1996). In a study with a large sample size of 2002 running associated injuries, the knee joint has been the most frequently affected (42.1%) and patellofemoral pain syndrome (PFPS) alone accounts for 46% (Taunton et al., 2002). PF (OA) was common in 20–30% of adults aged between 26–50 years with persistent PF pain (Collins et al., 2019). Health professionals such as physiotherapists use several assessment protocols to assess the disease impairments and to determine the effectiveness of treatment and the disease prognosis before, during and after the treatment. These can be broadly classified into subjective and objective protocols (Ateef, Kulandaivelan & Tahseen, 2016). A subjective questionnaire; the “Knee injury and Osteoarthritis Outcome Score (KOOS-PF) Patello Femoral”, a disease-specific questionnaire to measure knee problems with patellofemoral origin or along with osteoarthritis knee patients and KOOS-PF has a total of eleven items to comprehensively assess the patellofemoral origin of symptoms such as stiffness, pain and quality of life (http://www.koos.nu/).

Assessment of range of motion, muscle atrophy comes under objective examination. In terms of subjective evaluation, a well-translated, cross-culturally adapted and psychometrically validated questionnaire would provide a piece of comprehensive symptomatic information to the evaluator. Since TFJ and PFJ arthritis differ symptomatically and the target population is different as sports individuals commonly come across PFJ arthritis, a separate disease-specific KOOS-PF subscale was evolved from the KOOS long-form to subjectively evaluate the PFJ arthritis (http://www.koos.nu/). KOOS-PF measurement properties are well-validated along with adequate structural validity and patient responsiveness (http://www.koos.nu/; Crossley et al., 2018). Currently, the validation process is going on in seven languages (http://www.koos.nu/) and Arabic is one among them. To better serve Saudi Arab patients with patellofemoral pain syndrome, the original language KOOS-PF questionnaire need to be validated psychometrically in Saudi Arabia. This would allow better evaluations to be carried out because patients can better express their symptoms in their native language.

The primary objective of the paper was to cross-culturally translate and adapt the KOOS-PF to the Saudi dialect of the Arabic language. The secondary aim was to determine the measurement properties of the translated version such as internal consistency, test-retest reliability, floor-ceiling effect, inter-item correlation, the percentage of agreement and construct validity. 

## Materials and Methods

### Study protocol

The present validation process has been approved and with the permission from the original developer (dated 29/08/2018) of the instrument, University of Southern Denmark, Denmark. The process of translation of Arabic abstract can be found on the website (http://www.koos.nu/). KOOS-PF contains 11 questions out of which one is for symptom; nine for pain and one about quality of life. The exact phrase of questions is PF1. How severe is your knee stiffness after exercise? PF2. How often do you experience knee pain after stopping activity? PF3. How often does pain limit your activity? PF4. Rising from sitting (including getting out of the car). PF5. Kneeling PF6. Squatting PF7. Heavy household activities (including carrying and lifting) PF8. Hopping/jumping PF9. Running/jogging PF10. After sport and recreational activities PF11. Have you modified your sport or recreational activities due to your knee pain? Each question contains 5 options never to always (less disability to maximum disability); according to guidelines from the main developers of the KOOS, it is mandatory to mention the direction of scoring the data for better interpretation of results where the highest obtained values indicate no symptoms and lowest were the extreme symptoms. The method of scoring should be to calculate the mean score of the 11 items and divide by the maximum possible score for each item (i.e., 4), using the formula 100–[(mean score PF1-PF11)/4 * 100] (http://www.koos.nu/).

The present study has followed the Beaton et al. (2000) guidelines of the validation process. The religious-cultural activity (prayer/ ‘Salat’) has been taken into consideration while adapting the questionnaire as the Muslim population offer prayers five times every day and involves the knee joint activity in different positions of the prayer. Two translators have involved in Stage I of the process to translate the original (forward) to target language by (T1-informed clinical expert and T2-uninformed non-clinical expert), whereby the English version of KOOS-PF was first translated into the target dialect of the Arabic language. The Questionnaire was synthesized as an Arabic draft (T-12) after analysis of language discrepancies by the two translators (T1&T2) during stage II.  The translated questionnaire was back-translated to English separately by two blinded, Arabic-native English professors to create two English versions (BT1 & BT2) during stage III (Beaton et al., 2000).  All the suggestions were taken into consideration from the initial draft written by the first two translators and back-translation by two qualified English language experts and one Orthopedic surgeon, who could suggest technical discrepancies to produce a pre-final version during stage IV.  During the stage V, the pre-final version was then administered to twenty local Arabic-speaking citizens, who were suffering from anterior knee pain (twelve men and eight women) and checked for the flow of the language and understanding of the items related to their knee problem. The panel then considered the opinions of all these subjects and agreed to add Arabic words ‘Sujud’ and ‘Rakaa’ to the items wherever suitable. The word ‘Sujud’ refers to touching the forehead to the floor during (‘Salat’) prayers, (i.e., “prostrating” and the word ‘Rakaa’ refers to kneel sitting) to the item No. PF1(stiffness) “How severe is your knee stiffness after exercise/**prostrating**, **kneel sitting**?”; the word prayer to PF2 (Pain). “How often do you experience knee pain after stopping activity/prayer?”; item No. PF3 “How often does pain limit your activity/**prostrating**, **kneel sitting**?”; item No. PF4 “Rising from sitting” (including getting out of the car/ **prostrating**, **kneel sitting**); item No. PF5. “Kneeling” (**during prayers**); item No.PF6 “Squatting” (**during prayers**); item No.PF10. (pain) “After sport and recreational activities”, **prayers/ ‘Sujud’,** **‘Rakaa”**. The word ‘Salat’ refers to prayer added to item No.PF11. “Have you modified your sport or recreational activities and Salat (prayers) due to your knee pain?” After the subjects were convinced with the items, a final draft evolved as a complete pre-testing questionnaire (stage V). After all the above stages, the measurement properties were measured in target individuals with chronic anterior knee pain.

### Study sample

A total of 95 male subjects with anterior knee pain were selected in the present study. The inclusion criteria were patients aged between 32 to 66 years as the patellofemoral pain affects the young and middle-aged (Collins et al., 2019) commonly in whom sports/recreational/praying activity becomes the main reason for suffering the anterior knee pain (PFPS). The final draft of the questionnaire was explained in detail to the patients, including the average time required to fill in the self-reported form. The following exclusion criteria were implemented to prevent biased outcomes: presence of the other variant rheumatic diseases, severe inflammatory arthritis, knee problems other than anterior knee pain, low back pain, corticosteroid therapy as confirmed by Orthopedic Surgeon.

### Data collection

Patients were selected through a convenient sampling technique from the outpatient departments of three different hospitals in and around Al Majmaah city, KSA who were diagnosed by an Orthopedic surgeon, from 1st November 2019 to 31st January 2020. Patients who were eligible with the inclusion criteria were asked to participate in the study. All included patients were provided with informed written consent; they were then asked to complete the translated Arabic questionnaire on two different occasions, with a 48-hour gap in-between; to ensure that their answers were reliable. The questionnaire was explained to all patients with mention of its 11 items of KOOS-PF related to their anterior knee pain. Patients were given the questionnaire soon after the diagnosis of their knee condition. Among the 95 subjects who were asked to come back after 48 h to refill the questionnaire, 84 patients (88.4% compliance rate) responded. I chose the 48-hour time gap to avoid measuring the effects of prescribed drugs on pain; as well as to prevent recall bias in questionnaire administration. Subjective pain was measured using the numeric visual analog scale (VAS). The psychometric measurement properties of the translated version such as internal consistency, test-retest reliability, floor-ceiling effect, inter-item correlation, the percentage of agreement and construct validity were done using IBM-SPSS statistical (Version 24.0) software.

### Measurement of psychometric properties

#### Internal consistency

Cronbach’s Alpha (average correlation) psychometric statistical test was performed to measure the internal consistency of all the items of the questionnaire based on the initial data. Good internal consistency of the subscale was considered between 0.7 to 0.9, item will be removed if a value is less than 0.7 and excellent if the value is greater than 0.9 (Rattray & Jones, 2007; Juul et al., 2016).

### Test-retest reliability

The Intraclass correlation coefficient (ICC) (single measure) was measured to see the difference between the first and second (Test and retest) self-rated form for its reliability calculated with 95% confidence interval (Rattray & Jones, 2007; Juul et al., 2016). The two-way random effect model and an absolute agreement were utilized to measure the ICC. ICC value should be >0.80 for good test-retest reliability. Forty-eight hours (2 days) of time interval was opted for evaluating test-retest reliability to avoid the outcome bias due to treatment drug effect.

### Inter-item correlation matrix

This matrix within the subscale should be between 0.3 –0.8; anything less than 0.3 and more than 0.8 should be considered as redundant hence removed (Juul et al., 2015).

### Percentage of agreement

The percentage of the agreement was calculated in two ways, coefficient of variance (CV) in percentage (Simonsen, 1995) and minimal detectable change (MDC) in % (Lee et al., 2013). The CV was calculated with SD divided by mean which provides a ratio for variability. This ratio can be multiplied by 100 for percentile. The MDC could be calculated by two steps, the first measurement of error (SEM) by SD_Pooled_ $\sqrt{(1-r)}$; secondly measurement of MDC by 1.96* $\sqrt{2}$*SEM. Thirdly MDC value will be divided into mean and this value can be multiplied by 100 for percentile. Values less than 30% is acceptable and if values are less than 10% that is excellent.

### Construct validity

The construct validity was assessed at obtaining baseline data through Spearman correlations to check the relationship between the KOOS-PF scale and with the numeric visual analog scale (VAS). Standard values of more than 0.70 were considered strong, between 0.70–0.50 moderate and less than 0.50 weak for Correlation coefficients (Terwee et al., 2007).

### Floor and ceiling effect

The floor and ceiling effects were evaluated for the items of the KOOS-PF questionnaire. The obtained scores are statistically acceptable when less than 15% of the participants reporting the lowest or highest possible score (Roos et al., 1998). Ceiling (highest 100%) and floor (Lowest 0%) effect of each individual subscale has been observed individually in percentage (Roos et al., 1998).

## Results

Table 1 shows the percentage of participants who were less than 40 years 18(18.9%) and 77(81.1%) were more than 40 years of age with a mean age of 49.75 ± 9.87; mean and standard deviation of Body Mass Index 27.99 ± 3.94; mean and standard deviation of KOOS-PF score was 35.63 ± 12.25 respectively.

**Table 1:** Demographic and baseline data (*n* = 95).

	Mean (SD) Range
Males (%)	39 (41%)
Age ≤ 40 (Years)	18(18.9%)
Age ≥40 (Years)	77(81.1%)
Age	49.75(9.87) (32–66)
Body Mass Index (Kg/m^2^)	27.99(3.94) (21.5–36.4)
KOOS-PF	35.63(12.25) (13.9–77.3)

**Notes.**

SD Standard Deviation Kg/m^2^ kilogram per meter square KOOS-PF The Knee injury and Osteoarthritis Outcome Score Patello Femoral

Table 2 Cronbach’s alpha ∞ was measured/calculated based on the baseline data obtained from the eleven items of the KOOS-PF. Table 2 shows the Cronbach’s alpha ∞ 0.81 for eleven items of KOOS-PF Subscale. The obtained result 0.81 found between 0.7 to 0.95 which shows that the internal consistency between items was acceptable.

**Table 2:** Internal consistency of 11 items (*n* = 95).

Measurement property	Cronbach’s Alpha	Cronbach’s Alpha based on standardized items	No of items
Internal consistency	0.81	0.81	11

**Notes.**

KOOS-PF The Knee injury and Osteoarthritis Outcome Score Patello Femoral

Table 3 shows the Intra Class Correlation to evaluate the reliability. The value 7 or more than 7 is acceptable. A Two-way mixed model was used to draw the results. The single measure was 0.922 with the lower bound of 0.746 and the upper bound of 0.965 whereas the Average measure was 0.959 with the lower bound of 0.855 and the upper bound of 0.982. The values were statistically significant when the participants have filled the questionnaire after forty-eight hours of baseline data obtained. ICC value should be more than 0.80 for good reliability. The minimum gap of 48 hours has been maintained to avoid any drug effects which may alter the retest results.

**Table 3:** Intraclass correlation coefficient ICC (*n* = 84, 88.4%) of KOOS-PF subscale.

	Test and retest reliability	95% Confidence interval		Value	Significance *p* value
		Lower bound	Upper bound		
Single measure	0.922	0.746	0.965	37.13	<0.001
Average measure	0.959	0.855	0.982	37.13	<0.001

**Notes.**

ICC Intraclass Correlation Coefficient KOOS-PF The Knee injury and Osteoarthritis Outcome Score Patello Femoral

* *p*-value significance.

Table 4 shows the Baseline score mean (SD) 35.637 ± 12.25; Re-test score Mean (SD) 38.538 ± 12.03 and the mean difference between the baseline and a retest scale score was 2.9 with the test and re-test reliability of 0.959 and the standard error of measurement was 1.2.

**Table 4:** Test and re-test reliability and measurement error.

Subscale	Baseline score (Mean)SD	Re-test score (Mean)SD	Mean difference	ICC (95% CI)	SEM
KOOS-PF	35.637(12.25)	38.538(12.03)	2.901	0.959 (0.855–0.982)	1.2

**Notes.**

ICC Intraclass Correlation Coefficient SD Standard deviation CI Confidence Interval SEM standard error of measurement KOOS-PF The Knee injury and Osteoarthritis Outcome Score Patello Femoral

### Inter-item correlation matrix

Table 5 shows the Coefficient of variation (CV) as a percentage, of all items in the Arabic KOOS-PF. These CV values indicate that no item was precise but overall items in the Arabic KOOS-PF fell within the acceptable range (<30%).

**Table 5:** Coefficient of variation (CV) and Minimal detectable change % (MDC%) for all items in Arabic KOOS-PF (*n* = 95).

SNO	Construct	Coefficient of variation (CV)	Minimal detectable change (MDC%)
1	KOOS-PF	51.7%	(7.921/47.1)100 = 16.91%

**Notes.**

KOOS-PF The Knee injury and Osteoarthritis Outcome Score Patello Femoral

When the correlation analysis was done between all the items of the KOOS-PF subscale, all the values were found below 0.8. Minimum inter-item correlation found between PF3 and PF11 (0.318) whereas, the maximum inter-item correlation found between PF2 and PF8 (0.784). None exceeded the standard value of 0.8 which indicates a good inter-item relationship (Table 6).

**Table 6:** Inter-item correlation matrix.

	PF1	PF2	PF3	PF4	PF5	PF6	PF7	PF8	PF9	PF10	PF11
PF1	–										
PF2	0.327	–									
PF3	0.664	0.391	–								
PF4	0.349	0.513	0.349	–							
PF5	0.364	0.357	0.373	0.448	–						
PF6	0.479	0.400	0.320	0.337	.390	–					
PF7	0.541	0.386	0.446	0.362	.363	0.387	–				
PF8	0.399	0.784	0.322	0.376	.383	0.352	0.335	–			
PF9	0.584	0.361	0.379	0.355	.410	0.694	0.371	0.346	–		
PF10	0.336	0.376	0.498	0.398	.394	0.365	0.663	0.336	0.344	–	
PF11	0.539	0.320	0.318	0.467	.471	0.488	0.419	0.385	0.771	0.365	–

### Construct Validity

VAS has a moderate correlation with the KOOS-PF items (−0.568).

### Floor and Ceiling effect

Table 7 shows the floor (lowest) and ceiling (highest) values of individual items (questions) as there was no floor or ceiling effect for individual items in Arabic KOOS-PF (Table 7). The lowest floor effect opted was 4.7% for the item No. PF8 and the highest was 13.9% for the PF4. The lowest Ceiling effect opted was 2.2% for the item No. PF1 and the highest was 13.4% for the PF11.

**Table 7:** Mean, SD, item-total correlation along with floor (lowest ‘0’ score) and ceiling (highest ‘4’ score) effect of individual items in Arabic KOOS-PF (*N* = 95).

S No	Variable	Mean	Standard deviation	Item-total correlation	Floor effect	Ceiling effect
1	KOOS-PF1	2.4737	0.74151	0.510	6.0	2.2
2	KOOS-PF2	2.3789	0.78793	0.351	9.0	11.4
3	KOOS-PF3	2.6421	0.69826	0.383	12.2	9.0
4	KOOS-PF4	2.7579	0.78165	0.548	13.9	3.4
5	KOOS-PF5	2.8737	0.85355	0.508	11.9	10.4
6	KOOS-PF6	2.2947	0.84039	0.593	10.4	4.9
7	KOOS-PF7	2.5158	0.63352	0.451	5.5	3.4
8	KOOS-PF8	2.4842	0.87352	0.140	4.7	2.9
9	KOOS-PF9	2.6526	0.99753	0.675	7.65	12.7
10	KOOS-PF10	2.3053	0.68531	0.452	9.7	10.3
11	KOOS-PF11	2.9158	0.84631	0.732	8.3	13.4

**Notes.**

KOOS-PF The Knee injury and Osteoarthritis Outcome Score Patellofemoral

Floor and ceiling effect values are in percentage (%).

## Discussion

The validated Arabic KOOS-PF and its psychometric properties shows that it has high internal consistency, high test-retest reliability, acceptable floor and ceiling effect, the inter-item correlation matrix is within the acceptable range item-total correlation is in acceptable range, and moderate construct validity with VAS. All these findings encourage one to use this questionnaire in patellofemoral joint pain patients, particularly during repeated praying activity. There is no questionnaire available to date to assess the patellofemoral pain syndrome in Arabic patients.

KOOS-PF results are in line with the results of the Arabic version of full-length KOOS in which the internal consistency of pain construct Cronbach’s alpha was 0.87 (Alfadhel et al., 2018) whereas KOOS-PF Arabic was 0.81. When compared with the domain wise, KOOS Arabic was 0.91 for symptoms domain, pain domain was 0.87, activities of daily living were 0.88, sports and recreation was 0.92 and knee-related quality of life was 0.9. There were some similarities between KOOS-PF (11 item scale) and KOOS (42 item scale) like 3 domains of KOOS-PF are also found in KOOS i.e., symptoms, pain and quality of life. KOOS-PF has internal consistency of 0.81 which is less than the long-form KOOS Arabic version which is falling nearer to 0.87–0.92 of Arabic long-form KOOS subscales (Roos et al., 1998; Duncan et al., 2009; Alfadhel et al., 2018). KOOS-PF Arabic version (Cronbach’s alpha 0.81), pain domain (item No: PF2, PF3, PF4) and QOL domain were closer to KOOS-Short Form (12 items) which has internal consistency (pain) of 0.75–0.95, (QOL) 0.8. The results of inter-item correlation of KOOS-PF Arabic version (pain items PF5 vs PF4; PF6 vs PF1 & PF2; PF7 vs PF3; PF9 vs PF5; PF10 vs PF3 (Table 6) and QOL items PF11 vs PF4-7) are similar to (pain, 0.43 and QOL 0.44) original developer of 12 items KOOS-SF (Gandek et al., 2019a; Gandek et al., 2019b). This shows good internal consistency of the items in KOOS-PF. This KOOS-PF validation result’s Cronbach’s alpha was less than the original KOOS-PF developed version, but within the acceptable range (Roos et al., 1998; Duncan et al., 2009; Crossley et al., 2018). The Cronbach’s alpha of the original KOOS-PF was 0.86. The developer of the KOOS-Short Form (12 items) have recently stated that different short forms with diverse content provide domain specific joint impact scores (Gandek et al., 2019a; Gandek et al., 2019b) which supports the concept of Arabic KOOS-PF cultural adaptation and validation. One KOOS-Urdu validation study (Ateef, Kulandaivelan & Alqahtani, 2017) also supports this Arabic KOOS-PF stiffness (PF1) Cronbach’s alpha (0.81) where it was marginally higher than their symptom Cronbach’s alpha 0.788. The other two item domains (pain, 0.87 and QOL, 0.72) are supporting the results keeping the values within the standard psychometric values (Terwee et al., 2007). According to psychometric standard values, a good internal consistency is acceptable between 0.7 to 0.9 (Terwee et al., 2007). This KOOS-PF internal consistency (0.81) was within the normal range and like the other three studies, indicating good internal consistency/correlation between the items of the KOOS-PF Arabic version.

The average measure of the Intraclass correlation coefficient (ICC) of Arabic KOOS-PF was 0.959 which indicates an excellent test and re-test reliability. KOOS-PF intraclass correlation coefficient (ICC) was higher than the original KOOS-PF (0.86) (Crossley et al., 2018). The Intraclass correlation coefficient (ICC) of KOOS-PF was 0.959 and higher to the symptom domain 0.94 (corresponding to Arabic KOOS-PF1), pain domain 0.93 (corresponding to Arabic KOOS-PF2-10) and QOL domain 0.93 (corresponding to Arabic KOOS-PF11) of long-form of KOOS Arabic version (Alfadhel et al., 2018). The Intraclass correlation coefficient (ICC) of KOOS-PF was 0.959 and similar to the symptom domain 0.96 (corresponding to Arabic KOOS-PF1), lower to pain domain 0.978 (corresponding to Arabic KOOS-PF2-10) and similar to QOL domain 0.968 (corresponding to Arabic KOOS-PF11) in long-form of KOOS Urdu version of India (Ateef, Kulandaivelan & Alqahtani, 2017).

Visual analog scale (VAS) has a moderate correlation with the KOOS-PF items (−0.568). The correlation >0.5 is acceptable as per the psychometric properties (Terwee et al., 2007). The long-form KOOS Arabic version construct validity of pain, symptom and QOL were −0.71, −0.59 & −0.64 (Alfadhel et al., 2018) which supports the Arabic KOOS-PF when correlated with VAS. The Minimal detectable change (MDC%) for Arabic KOOS-PF was 16.91%, which was acceptable if less than 30% (Lee et al., 2013) and the long-form KOOS Arabic version MDC% for pain was13.9%, symptom 14.25% and for QOL 12.57%, respectively (Alfadhel et al., 2018). None of the items of Arabic KOOS-PF have attained the floor or ceiling effects. All the obtained values were within the acceptable standard percentage,15% (Roos et al., 1998) i.e., participants (more than 15%) were not selected lowest ‘0’ and highest value ‘4’. These results were well supported by the original KOOS-PF questionnaire done by Crossley K. M and group in 2018 where they have also found no ceiling and floor effect (Crossley et al., 2018).

### Strength and limitations

The strength of this study is the only available Saudi dialect of Arabic questionnaire to evaluate the patellofemoral pain syndrome in the Saudi Arabian Muslim populace, which is well adapted based on the religious practice.

Duration between test and retest for reliability is too short but the author decided to use short interval to avoid any analgesic effect of the drug. Author used only the male subjects in the validation process as the male therapists are not allowed to treat/evaluate female patients in the hospitals of Saudi Arabia hence, the results may not apply to female patients. Future studies should attempt to measure this property in Saudi female patients. No validation studies of KOOS-PF have been done either in any other Arabic dialect or language other than the original English KOOS-PF version to support the results. The responsiveness property was not measured and intends to measure in the future by the same author.

## Conclusion

The Knee injury and Osteoarthritis Outcome Score (KOOS-PF) Patello Femoral questionnaire has obtained acceptable measurement properties, hence is sufficiently reliable, valid and free of the ceiling and floor effects to be used to evaluate the patellofemoral problems in Saudi Arabian anterior knee pain patients.

##  Supplemental Information

10.7717/peerj.9323/supp-1 Supplemental Information 1 Patient data used for the analysis of measurement properties

10.7717/peerj.9323/supp-2 Supplemental Information 2 Blank questionnaire

## Additional Information and Declarations

### Data Availability

The following information was supplied regarding data availability: The raw data are available in a Supplemental File.
