# Supplementary material for: Measurement properties of the Knee Injury and Osteoarthritis Outcome Score Patello-Femoral questionnaire in Saudi Arabians
Source: PeerJ. 2020 Jul 31;8:e9323. doi: 10.7717/peerj.9323 (PMC7397982; doi:10.7717/peerj.9323)
Supplement: Supplemental Information 2 [file peerj-08-9323-s002.pdf]

# Adaptation and Measurement Properties of Arabic Version of Knee injury and Osteoarthritis Outcome Score Patello Femoral Questionnaire among Saudi Arabian Muslim Populace.

* Mahamed Ateef

* Assistant Professor, Department of Physical therapy & Health Rehabilitation, College of Applied Medical Sciences, Majmaah University, Al Majmaah, KSA-11952.

Abstract

Objective: The aim of this study was to translate, adapt and psychometrically validate the Knee injury and Osteoarthritis Outcome Score (KOOS-PF) Patello Femoral from English to Arabic language of Saudi Arabian dialect. **Methods:** KOOS-PF was cross-culturally translated from English to Saudi Arabic based on Beaton et al guidelines. Next, 95 patients with chronic knee pain of patellofemoral origin were administered the questionnaire to determine reliability, with a 48-h rest period between test and retest. Internal consistency, agreement percentage (CV&MDC), inter-item correlation, intraclass correlation coefficient (ICC), and ceiling and floor effects were calculated to validate the Arabic KOOS-PF. Spearman's correlation was used to compare the KOOS-PF subscale with a visual analog scale(VAS) for construct validity. **Results:** Cronbach's alpha and test–retest reliability were good and excellent (∞>0.81; ICC>0.95). None of the items showed a floor or ceiling effect and the minimal detectable change was within acceptable range (< 30%). The KOOS-PF subscale showed a moderate correlation with VAS for its construct validity. **Conclusion:** The Arabic dialect of KOOS-PF is reliable and valid to be used to evaluate isolated knee pain of patellofemoral origin in patients with chronic knee patients.

Keywords: Knee, Reliability, Validity, Psychometric

Version LK 1.1: On November 12, 2019 the Arabic (Saudi Arabia) KOOS-PF was update to LK 1.1 with the following changes: In order to better cross-culturally adapt the questionnaire, question PF1-6 and PF10-11 have had additional examples added.

إصابة في الركبة والتهاب المفاصل – مفصل الركبة الرضفة الفخذية

تاريخ اليوم: ____________________ تاريخ الميلاد______________________

اسم____

يسأل هذا الاستطلاع عن رأيك حول ركبتك .هذه المعلومات سوف تساعدنا على الحفاظ وتتبع كيف تشعر حول ركبتك ومدى كنت قادرا على القيام المعتاد بالأنشطة الخاصه بك.

يرجى الإجابة على كل سؤال عن طريق وضع علامة في المربع المناسب، مربع واحد فقط لكل منهما سؤال.
إذا كنت غير متأكد من كيفية الإجابة على سؤال، يرجى إعطاء أفضل إجابة لك.

التيبس

السؤال التالي يتعلق بكمية الصلابة المشتركة التي واجهتها خلال الأسبوع الماضي في ركبتك .الصلابة هي الإحساس بالقيود أو البطء
في سهولة التي تتحرك مفصل الركبة.

PF1. ما مدى شدة ركبتك بعد التمرين/ سجود, راكع ؟

لا شيء	خفيف	معتدل	شديد	اقصي مدي
☐	☐	☐	☐	☐

الالم

الأسئلة التالية تتعلق بألم الركبة خلال الأسبوع الماضي.

PF2. كم مرة تعاني من آلام في الركبة بعد توقف النشاط/ صلاة/ سجود, راكع ؟

أبدا	شهريا	أسبوعيا	يوميا	دائما
☐	☐	☐	☐	☐

PF3. كم عدد المرات التي تحد فيها الألم من نشاطك/ سجود, راكع ؟

أبدا	شهريا	أسبوعيا	يوميا	دائما
☐	☐	☐	☐	☐

ما هو مقدار آلام في الركبة التي واجهت في الأسبوع الماضي خلال بعد الأنشطة؟

- يرجى إعطاء أفضل إجابة يمكنك، حتى لو كنت غير متأكد من عنصر
- إذا لم تكن قد فعلت هذا النشاط بسبب نصيحة طبية أو ألم، يرجى وضع علامة " أقصى"

PF4. ارتفاع من الجلوس (بما في ذلك الخروج من السيارة/ سجود, راكع).

لا شيء	خفيف	معتدل	شديد	اقصي مدي
☐	☐	☐	☐	☐

PF5. الركوع خلال الصلوات.

لا شيء	خفيف	معتدل	شديد	اقصي مدي
☐	☐	☐	☐	☐

PF6 . القرفصاء أثناء الصلاة.

لا شيء	خفيف	معتدل	شديد	اقصي مدي
☐	☐	☐	☐	☐

PF7. الأنشطة المنزلية الثقيلة (بما في ذلك الحمل والرفع)

لا شيء	خفيف	معتدل	شديد	اقصي مدي
☐	☐	☐	☐	☐

PF8. النط / القفز

لا شيء	خفيف	معتدل	شديد	اقصي مدي
☐	☐	☐	☐	☐

PF9. الجري / الركض

لا شيء	خفيف	معتدل	شديد	اقصي مدي
☐	☐	☐	☐	☐

PF10 . بعد الرياضة والأنشطة الترفيهية/ سجود و/راكع .

لا شيء	خفيف	معتدل	شديد	اقصي مدي
☐	☐	☐	☐	☐

جودة من حياة

السؤال التالي يتعلق بنوعية حياتك خلال الأسبوع الماضي

PF11. هل عدلت الرياضة أو الأنشطة الترفيهية و صلاة بسبب آلام الركبة.

بسبب آلام الركبة ؟

لا على الإطلاق	تعديل خفيف	معتدل إلى حد ما	معتدل بشدة	تعديل كلي
☐	☐	☐	☐	☐
